# Simple imputation methods were inadequate for missing not at random (MNAR) quality of life data

**DOI:** 10.1186/1477-7525-6-57

**Published:** 2008-08-04

**Authors:** Shona Fielding, Peter M Fayers, Alison McDonald, Gladys McPherson, Marion K Campbell, the RECORD study group

**Affiliations:** 1 Department of Public Health, University of Aberdeen, UK; 2 Department of Cancer Research and Molecular Medicine, Faculty of Medicine, Norwegian University of Science and Technology, Trondheim, Norway; 3 Health Services Research Unit, University of Aberdeen, UK

## Abstract

**Objective:**

QoL data were routinely collected in a randomised controlled trial (RCT), which employed a reminder system, retrieving about 50% of data originally missing. The objective was to use this unique feature to evaluate possible missingness mechanisms and to assess the accuracy of simple imputation methods.

**Methods:**

Those patients responding after reminder were regarded as providing missing responses. A hypothesis test and a logistic regression approach were used to evaluate the missingness mechanism. Simple imputation procedures were carried out on these missing scores and the results compared to the actual observed scores.

**Results:**

The hypothesis test and logistic regression approaches suggested the reminder data were missing not at random (MNAR). Reminder-response data showed that simple imputation procedures utilising information collected close to the point of imputation (last value carried forward, next value carried backward and last-and-next), were the best methods in this setting. However, although these methods were the best of the simple imputation procedures considered, they were not sufficiently accurate to be confident of obtaining unbiased results under imputation.

**Conclusion:**

The use of the reminder data enabled the conclusion of possible MNAR data. Evaluating this mechanism was important in determining if imputation was useful. Simple imputation was shown to be inadequate if MNAR are likely and alternative strategies should be considered.

## Background

Missing data are a common occurrence in any area of research, and are especially problematic in quality of life (QoL) studies. Data may be missing for a variety of reasons. If these reasons relate to the QoL of the patient, the missingness is informative. Simply excluding those with missing data from the analysis ("complete case analysis"), will bias the results if those who did not respond had significantly lower (or higher) QoL scores than those who did respond.

Rubin [1] defines three main mechanisms of missing data: missing completely at random (MCAR), missing at random (MAR) and missing not at random (MNAR). MCAR requires very strong assumptions. An observation is said to be MCAR if the missingness is independent of all observed and unobserved (i.e. previous, current and future) QoL assessments [2]. For example a patient may simply forget to post the questionnaire back. Observations can also be MCAR if the missingness only depends on values of fixed covariates that are measured prior to treatment assignment – often termed covariate-dependent dropout. For example, if elderly patients were less likely to respond, missingness would be dependent on age group.

A more relaxed assumption about the missing data mechanism is missing at random (MAR), where missingness is independent of all unobserved (missing or future) QoL values, although it may be dependent on the observed values. The "observed values" may comprise a baseline measure of QoL or a previous assessment and any appropriate covariates.

A process that is neither MCAR nor MAR is called missing not at random (MNAR). MNAR occurs if missingness depends not only on the observed data but also on the unobserved (missing) values. An example is that a person with reduced QoL due to side effects of treatment may be less likely to return the questionnaire. The missing value depends on the unobserved QoL scores and the missingness mechanism is informative.

Many investigators have explored approaches to determine the mechanism of missingness. They have either generated artificial datasets using simulation techniques [3], or have made use of existing datasets in which missing data were then artificially created [4]. These procedures are potentially misleading: the missing patterns are predetermined and pre-specified, and usually the performance of the various tests can be anticipated through the known mechanism that was used to generate the samples.

One approach to deal with missing data is simple imputation, which is the process whereby a single estimated value for the missing observation is obtained, thereby enabling standard statistical methods to be applied to the augmented data set. Various methods can be implemented to impute the missing data. However, the accuracy of imputation cannot normally be determined, as the true values are not known. Various authors have explored the potential accuracy of imputation methods by artificially removing data from a dataset and treating it as missing [3-5]. This is a circular argument, as noted above, because the data are either removed at random or according to some known and pre-specified pattern. In practice, the major analytical problem is that one does not know the exact missing mechanism.

Engels and Diehr [6] noted the need to use data with real missing patterns, and attempted to overcome these problems by using a dataset where a value was observed after one or more missing values had occurred; the observed value was treated as the true value for the missing data at the preceding time points. Various imputation methods were applied for the missing values, and the results compared against the observed value to assess accuracy of the imputation methods. As Engels and Diehr [6] comment, "this analysis hinges on the similarity of a known value following a string of missing values to other observations that are missing at that same time."

Poor compliance with collecting QoL data is a well-recognised problem in clinical trials. In an attempt to minimise the level of missing data, the Health Services Research Unit (HSRU) at the University of Aberdeen makes strenuous efforts to recover QoL data. When QoL questionnaires are not returned, HSRU not only issues repeated reminders (including telephone contact), but in addition offers to interview patients by telephone. Therefore, a proportion of patients who initially had missing data – as would have been the case in most clinical trials – then have "true" values which were subsequently recovered. This provided a unique opportunity to investigate the performance of tests for identifying missing data mechanisms and methods of imputation, because the results could be evaluated against the data that was later recovered.

## Methods

### The dataset

The RECORD trial was a randomised placebo-controlled trial of daily oral vitamin D and calcium in the secondary prevention of osteoporosis-related fractures in older people [7]. Patients' QoL was assessed by postal questionnaire at 4, 12, 24, 36 and 48 months. The four month data were considered the "baseline" measure as QoL for many patients at entry to the trial would be artificially low while they were being treated in hospital for their primary fracture. The questionnaire included the five items of the EuroQoL EQ5D [8], and the 12-item SF12 questionnaire [9]. The EQ5D produces a single QoL score, and the SF12 gives two summary scores, the physical and mental component scores (PCS and MCS). The results for EQ5D data are presented here. At each occasion, if a participant did not return the questionnaire within two weeks, up to two reminders were issued (two weeks apart). Patients who returned the questionnaire without needing a reminder were considered 'immediate-responders', while those that returned a questionnaire after one or two reminders provided 'missing yet known' data, and were termed 'reminder-responders'. In the analyses that follow, the scores obtained for reminder-responders were regarded as missing – what they would have been in some clinical studies.

### Identifying the missing data mechanism

#### Hypothesis tests

The pattern of missing data can be described as either "terminal", when no further observations were made on a patient after a set of complete observations, or "intermittent", in which case one or more observations for a patient were missing before a subsequent observation was observed. It was possible for a patient to have a mixed pattern, with a period of intermittent dropout followed by terminal dropout.

There are a number of hypothesis tests that can be carried out to test the assumption of MCAR. Little [10] developed a test based on the means of the variable of interest under the different missing data patterns (including intermittent and terminal missingness). Alternative hypothesis tests have been suggested by Diggle [11], Ridout [12] and Listing and Schlittgen [13], all requiring terminal missingness. Diggle [11] used an approach which tests whether the subset about to dropout are a random sample of the whole population. Ridout [12] adopted a similar approach to Diggle by utilising logistic regression. Listing and Schlittgen [13] proposed a test based on means. These alternatives to Little [10], will be less optimal in a situation where intermittent missingness is evident. Restricting the analysis to only those showing a terminal missingness pattern would cause a loss of information. Since RECORD contained intermittent missingness, Little's test was used to illustrate a hypothesis test for MCAR.

Little's test of MCAR versus MAR [10] is based on the rationale that if the data are MCAR then at each time point the calculated means of the observed data should be the same irrespective of the pattern of missingness. For example, it should not matter whether the previous assessment was observed or not, nor whether the one before that was observed. If the data are not MCAR, the mean scores will vary across the patterns. Consider a study with *J *measurements of QoL. Let *P *be the number of distinct missing data patterns (*R*_*i*_) where *J*^{*p*} ^is the number of observed variables. *n*^{*p*} ^is the number of cases with the *p*^th ^pattern and ∑*n*^{*p*} ^= *N*. Let *M*^{*p*} ^be a *J*^{*p*} ^*x J *matrix of indicators of the observed variables in pattern *P*. The matrix has one row for each measure present consisting of (J-1) zero's and one 1 identifying the observed measure.

${\bar{Y}}^{\left\{p\right\}}$ is the *J*^{*p*} ^*x*1 vector of means of the observed variables for pattern *p*, $\hat{\mu }$ is the maximum likelihood (ML) estimate of the mean of *Y*_*i *_and $\hat{\sum }$ is the maximum likelihood estimate of the covariance of *Y*_*i*_. The ML estimates assume the missing data mechanism is ignorable. ${\hat{\mu }}^{\left\{p\right\}}=M^{\left\{p\right\}}\hat{\mu }$ is the *J*^{*p*} ^*x*1 vector of ML estimates corresponding to the *p*^*th *^pattern and Σ˜=NN−1M{p}ΣˆM{p}' is the corresponding *J*^{*p*} ^*x J*^{*p*} ^covariance, matrix with a correction for degrees of freedom. Little's proposed test statistic when Σ is unknown, takes the form

$$X^{2}=\sum_{p=1}^{P}n^{\left\{p\right\}}\left({\bar{Y}}^{\left\{p\right\}}-{\hat{\mu }}^{\left\{p\right\}}\right)'{\tilde{\Sigma }}^{\{p\}-1}\left({\bar{Y}}^{\left\{p\right\}}-{\hat{\mu }}^{\left\{p\right\}}\right).$$

This test statistic is asymptotically chi-squared with (Σ *J*^{*p*} ^- *J*) degrees of freedom.

#### Logistic regression

Fairclough [14] described an approach to determine the missing data mechanism using logistic regression. The process investigates the missingness mechanism from a cross-sectional standpoint, each time point assessed in turn. Those people who did not respond were excluded from these analyses. An indicator variable was created to identify those patients who responded without the need for a reminder (immediate-responders) and those which were reminder-responders. The first step identified covariates that predict the occurrence of missing observations (reminder-response). Differences between the two groups with respect to a number of covariates were explored with t-tests and chi-squared tests. Logistic regression analyses were used to model the probability of missing an assessment. Identified covariates were forced into the model and the observed QoL scores tested as to whether they also contributed to the prediction of missingness [14], as indicated by a reduction in deviance (change in -2*log likelihood). The statistical significance of this reduction in deviance was assessed by comparing it to an appropriate chi-squared distribution (χ^2^_1_).

The advantage of this approach in our setting was the incorporation of the reminder data. A subset of data containing only responders was utilised. The data obtained by reminder was regarded as missing. Initially the process outlined above was carried out assessing whether the covariates and observed QoL were significant predictors of missingness (reminder response). Since the current QoL scores were known, the significance of these to predict missingness (reminder-response) could be assessed. If these scores were found to be statistically significant, the process suggests that data were potentially MNAR.

### Simple imputation

#### Methods of imputation

Simple imputation methods use information from other people (cross-sectional), or information pertaining to the person whose QoL data were missing (longitudinal) [15]. Longitudinal methods include last value carried forwards (LVCF), next value carried backwards (NVCB), last-and-next (LaN – average of last value and next value), average available (Avg), average of previous (prev) and average of future (post). Regression can also be carried out utilising other observed QoL scores (regP) or suitable covariates (RegC) or both together (regP2). Some of these methods cannot be utilised at every time point, e.g. *LaN *cannot be used to impute the 48 month scores since there is no 'next' value. Cross sectional methods include mean imputation, regression and hot-decking (random selection from those observed). A disadvantage of regression methods is that people with the same covariate set will have an identical imputed value. This can lead to the variance of the imputed data being artificially small, producing inappropriate standard errors, leading to inflated test statistics and falsely narrow confidence intervals and inappropriate *p*-values in any subsequent analysis [14,15].

A newer method not considered here is that of multiple imputation [14]. This procedure imputes a number of values for the missing data incorporating both the variability of the QoL measure and the uncertainty surrounding the missing observation. Each dataset is then analysed and the results combined. The focus of this paper however, is the adequacy of simple imputation.

#### Assessing accuracy of methods

The reminder-responses were regarded as missing and imputed using the methods explained above. The accuracy of these methods was then assessed by comparing imputed scores to the actual observed scores (of the reminder-responders), using a bias measure and proportionate variance (PV):

$$\begin{array}{l} Bias=\sum \left(y-\hat{y}\right)/m\\ PV=\mathrm{var}\left(\hat{y}\right)/\mathrm{var}\left(y\right) \end{array}$$

Where $\hat{y}$ is the imputed value, *y *is the actual value and *m *is the number of missing values. A positive *Bias *indicated that on average the imputed value underestimated the true QoL value. The *PV *is the ratio of the observed variance to the true variance and assesses the under-dispersion for each method. A *PV *of one indicates that the variance of the imputed values is equal to that of the true values. A *PV *of less than one implies underestimation of the true variance. The bias and PV were calculated for each patient and then an average was taken across all patients. To produce confidence intervals (CIs) for each of the accuracy estimates, the bootstrapping technique [16] was used within the statistical package STATA.

## Results

### Description of dataset

The RECORD trial recruited 5,292 patients, with characteristics shown in Table 1. The majority were female (85%), and most lived in their own home prior to (88%) and after (86%) the index fracture. The recruiting fracture was less than 90 days before recruitment for 82%, and 94% could walk outdoors unaccompanied. Recruiting fractures were in the arm (62%) or leg/hip (38%). Patients aged over 70 were eligible and 13% of those recruited were 85 and over. At four months, the proportion of deaths was larger in the older age group (85+).

**Table 1:** Patient characteristic of study population (N = 5292)

			**Percentage with score available at 4 m**		**Percentage without score available at 4 m**		
		**All Patients Number (%)**	**No reminder**	**After reminder**	**Not returned**	**Absent or withdrawn**	**Dead**
Age group	70–74	1917 (36)	40	37	29	29	12
	75–79	1665 (32)	33	31	31	30	18
	80–84	1030 (19)	17	19	23	24	33
	85+	680 (13)	10	13	17	17	36
Sex	Male	811 (15)	16	13	15	13	31
	Female	4480 (85)	84	87	84	87	69
Type of recruiting fracture	Proximal femur	904 (17)	16	17	20	18	47
	Other leg and pelvic	1130 (21)	22	20	21	20	17
	Distal arm	1846 (35)	36	35	31	36	20
	Other arm	1403 (27)	26	28	28	25	17
	Other	9 (<1)	0	0	0	0	0
Locomotor ability (Walk unaccompanied)	Yes	4979 (94)	95	93	93	93	85
	No	300 (6)	5	7	7	7	15
Time since recruiting fracture	≤ 90 days	4331 (82)	81	84	82	86	73
	> 90 days	961 (18)	19	16	18	14	27
Residence type *prior *to recruiting fracture	Own home	4628 (88)	89	86	84	87	78
	Sheltered housing	538 (10)	9	12	13	11	11
	Other	126 (2)	2	2	3	2	11
Residence type *after *recruiting fracture	Own home	4555 (86)	88	85	82	85	74
	Sheltered housing	531 (10)	9	11	12	10	11
	Other	206 (4)	3	4	6	5	15
Marital status	Single	348 (7)	7	6	7	6	7
	Married	2069 (40)	42	36	32	39	25
	Divorced	222 (4)	4	5	5	2	2
	Widow(er)	2634 (50)	47	52	56	52	65

Table 2 shows the number of EQ5D assessments at each time point. The number of questionnaires sent at each assessment reduces for two reasons. Firstly, not all patients were followed up after two years. Only those which were recruited early on in the trial were followed up for longer. These patients continued to be followed up until those recruited later had reached the two year assessment. Once all recruited patients were followed up for at two years, follow up stopped and no further data were collected. At 36 months, only 3,663 patients were followed up and this reduced further to 1,629 patients at 48 months. Secondly, some patients withdrew from the trial or died. The proportion of those sent questionnaires that provided valid QoL scores with or without reminder varied from 79% at 4 months to 86% at 48 months. Of those completing forms, 20% to 26% were reminder-responders. Overall, more than half of the data initially missing were recovered by the reminder system.

**Table 2:** Number (%) of EQ5D scores at each follow up point

	**Month of assessment**				
	**4**	**12**	**24**	**36**	**48**
**EQ5D score (no reminder)**	2908 (59)	2648 (62)	2511 (67)	1670 (69)	661 (69)
**EQ5D score (after reminder)**	999 (20)	840 (20)	693 (18)	406 (17)	162 (17)
**Not returned**	1042 (21)	763 (18)	561 (15)	338 (14)	138 (14)
**Total sent**	4949 (100)	4251 (100)	3765 (100)	2414 (100)	961 (100)
**Total available for follow up**	5292	5292	5292	3663	1629
**Not Sent**	343 (6)	1041 (20)	1527 (29)	1249 (34)	668 (41)
**Proportion of responders who did so by reminder**	26%	24%	22%	20%	20%

### Identifying the missing data mechanism

#### Hypothesis tests of MCAR

Considering data from the first three time points, Little's test statistic was X^2 ^= 133.75 (9 df) with p < 0.001. The data were restricted to those patients who responded at each of the first three time points (N = 2606) and data collected by reminder was set to missing. In this situation Little's test statistic was X^2 ^= 39.6 (9 df) with p < 0.001. Therefore, there was evidence against MCAR, suggesting that QoL impacted on whether or not a patient responded with or without the need for reminder.

#### Logistic regression

This section deals with responders only and the reminder-responders were regarded as missing. Using logistic regression at 12 months the covariates found to be significant predictors of missingness were gender, locomotor ability, residence type prior to fracture and marital status; at 24 months -gender, age group, locomotor ability and type of recruiting fracture; while at 36 months – age group and marital status; finally at 48 months – locomotor ability and time since recruiting fracture.

The change in deviance was used to determine whether the previous QoL score was a significant predictor having adjusted for covariates (Table 3). Previous QoL was defined as the most recent known QoL score prior to the time point of interest. The change in deviance was significant at 12 and 24 months. This indicated that, after adjusting for covariates, previous QoL remained important in modelling the probability of missing assessment. The null hypothesis of MCAR was rejected at 12 and 24 months. At 36 and 48 months there was insufficient evidence to reject the possibility that missingness was MCAR.

**Table 3:** Log-likelihood's for models 1–4

	**Month of assessment**			
	**12**	**24**	**36**	**48**
**Log-Likelihood**				
**L_1_: **MCAR fixed covariates	-1680.3	-1411.6	-846.3	-350.8
**L_2_: **MAR fixed covariates + previous QoL	-1673.9	-1409.5	-845.7	-350.8
**L_3_: **MNAR fixed covariates + current QoL	-1669.4	-1406.1	-843.2	-350.7
**L_4_: **MAR fixed covariates + previous QoL + current QoL	-1669	-1406.1	-843	-350.7

**Change in log-likelihood**				
-2*(L_1 _– L_2_) – test of MAR	12.8*	4.2*	1.2	0
-2*(L_1 _– L_3_) – test of MNAR	21.8*	11.0*	6.2*	0.2
-2*(L_2 _– L_4_) – test of MNAR	9.8*	6.8*	5.4*	0.2

* significant change, p < 0.05

In normal circumstances the investigation would stop at this point, because in most trials the true current score, *x*_*c*_, is not available for the "missing" group. However, using data collected by reminder the process was continued. Table 3 shows the log-likelihoods for model 3 (covariates + current QoL) and model 4 (covariates + previous and current QoL). After adjusting for both covariates and previous QoL, at 12, 24 and 36 months the current QoL was significant in the model, suggesting there was evidence of MNAR data. At 48 months there was no evidence that current or previous QoL were important in the model – but, at this time our sample size was substantially depleted.

Another question of interest was whether the non-responders were in any way different to the reminder-responders. A similar process was undertaken as above. The non-responders differed in one or two covariates at each time point but having adjusted for this, their previous score was not a significant predictor. Thus, there was no evidence that the previous QoL experience differed between the non-responders and the reminder-responders at a given assessment. This gave confidence that the reminder-responders were perhaps similar to the non-responders.

### Imputation of reminder-responder scores

**Figure 1:**
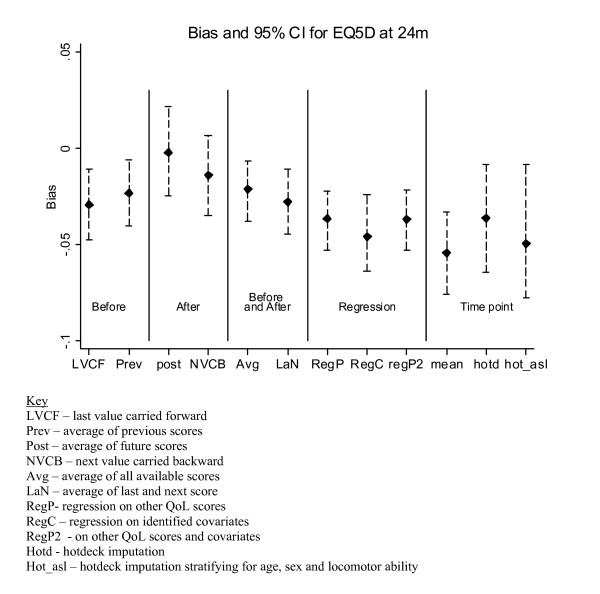
Bias results of EQ5D imputation at the 24 month follow up.

**Figure 2:**
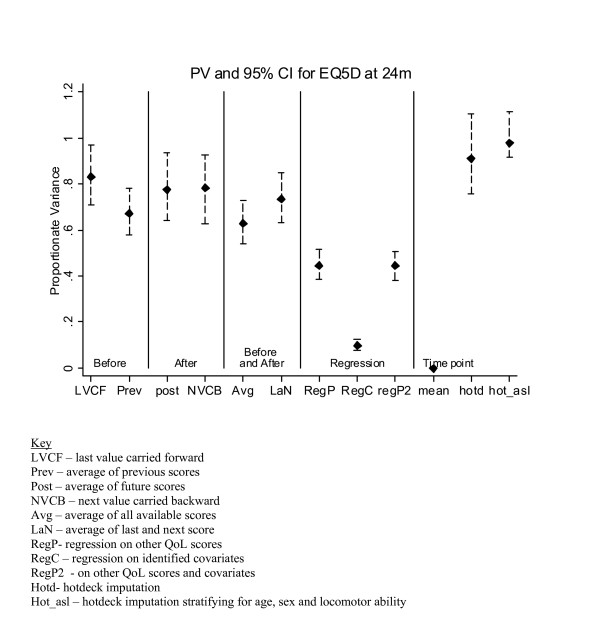
*PV *results of EQ5D imputation at the 24 month follow up.

Results for the imputed data were compared with the actual data and the 24 month data are presented in Figures 1 and 2. Figure 1 shows that at 24 months the smallest bias occurred with the *post *method (b = -0.002), while second smallest was *NVCB *(b = -0.014). The bias was significantly greater for the regression and cross-sectional approaches. At 4 and 12 months (data not shown), the *average *and *NVCB *were the best methods in terms of bias. At 36 months, none of the procedures provided a sufficiently accurate estimate and the bias was greater than -0.04. The number of procedures applicable at 48 months was reduced with the regression based on baseline characteristics showing the smallest bias (b = -0.004).

Figure 2 shows the best *PV *value for the 24 month data occurred with the hotdecking methods, which was perhaps expected since these methods impute using random selection from the immediate-responders. Hotdecking with stratification was the best of the two (PV = 0.979). The 'after' methods of *post *and *NVCB *had slightly lower *PV*, just under 0.8. The three *regression *procedures were very poor at preserving the variance. Since the same value is imputed for all missing values using the 'mean' methods, there was no variation in the imputed values, which would have a big impact on any subsequent tests and *p*-values.

At other time points (data not shown), where applicable, *NVCB *showed reasonable *PV*. The regression methods were consistently poor at preserving the variance. The *hotdeck *with stratification procedure was reasonably good at maintaining the variance at all time points (*PV *ranged from 0.87 to 1.27). By nature of the hotdecking procedure it is expected that the variance of the imputed values would be the same as that of the true values. Although the observed PV was not equal to one, the 95% CI did include the desired value of one, suggesting that the sample being imputed was similar to that from which values are being selected.

In general, for the RECORD trial methods involving QoL scores surrounding (and in particular those after) the point of imputation were the most accurate in terms of bias and at preserving the variance.

## Discussion

Identification of the correct mode of missingness and most appropriate method of imputation can make a large impact on the analysis of clinical trials. The sensitivity of different analyses depends on the proportion of missing assessments and the strength of the underlying causes for missing data [17]. The undesirable effect of missingness on bias and power increases with the severity of non-randomness as well as the proportion of missingness [18].

Little's test [10] for MCAR showed evidence against MCAR in favour of MAR between responders and non-responders and also between the immediate- and reminder-responders. The logistic regression approach showed on the whole, at each of 12, 24 and 36 months, after adjusting for the required covariates, both the previous and current QoL scores were significant predictors of missing assessment (response by reminder). This implied there was evidence of MNAR data at 12, 24 and 36 months. It is possible that the "reminder-responders" may differ from the persistent non-responders, but the analyses found no evidence of this in terms of previous QoL scores. This approach using data collected through reminders has provided an indication of MNAR, with the rationale that reminder-responders were more likely to be similar to the non-responders than the immediate-responders.

It should be noted that data collected through reminders has been assumed to be equivalent to that collected immediately. However, data collected via reminder are actually reflecting a time two (or four) weeks later than the original assessment time. This may bias the recovered data, but for the purposes of this investigation we assumed it to be comparable to data collected without the need for reminder.

The missingness mechanism was identified as potentially MNAR, but was simple imputation adequate? The results suggested that for the RECORD study the missing QoL scores could be imputed using assessments close to the point of imputation. In many QoL studies the assessments are taken at frequent intervals and the correlations between successive measurements may be high. Those imputation methods that focus on within-patient assessments close in time to the missing values are likely to be most effective. The population based methods assume the data are either MAR or MCAR. Since the data in this study were most likely MNAR, it is not surprising that these imputation methods were less accurate.

Data that are MNAR may depend on current and future observations, thus methods that utilise this data are intuitively going to be more accurate than those based on previous measures. *NVCB *and *post-average *showed the smallest bias. Although, the methods involving previous scores are useful, they can never be entirely accurate in the presence of MNAR. The methods of *NVCB *and *post-average *may not be practical as they are dependent on future QoL scores being available, which will only happen when missingness is intermittent. Often, in trials, the final assessment is the main focus and no future data are available to inform the imputation. Only methods using 'before' data are available, and these methods have shown to provide greater bias, suggesting that simple imputation is inadequate in the presence of MNAR data.

Limitations of this study are that the data are from a single trial, involving older people, and the studied disease is perhaps not typical of studies involving QoL assessments. However, our results agree with Engels and Diehr [6], despite being from a different disease, different country and for different QoL outcomes. We infer from this that the results may perhaps be generalisable.

If imputation procedures are to be employed, researchers need to be confident of their accuracy. One apparent advantage of imputation is that, once missing values have been filled in, standard methods of analysis can be undertaken on this augmented dataset comprising the observed and the imputed values. However, imputed values cannot be regarded as the same as if the full data has been observed. Although some summary statistics such as means and medians may not be distorted, the corresponding standard deviations may be shrunk and this will have consequences for the subsequent calculation of the confidence intervals [15]. This consequence of simple imputation is present whatever the missingness mechanism and provides a major disadvantage against the use of simple imputation procedures, even if one can assume the unlikely scenario of MCAR data.

Although the imputation may overestimate the true values in the reminder group, it may still bring the overall scores closer. What matters most is minimising the bias in treatment comparisons. An investigation into the effect of the different methods of imputation on the treatment effects forms the basis of future work.

During RECORD the issuing of reminders substantially increased the number of included patients, with corresponding gains in statistical power and the assurance of reducing the bias by avoiding the need for imputation. The reminder system entails extra resources. However, in any study having as much data as possible for analysis is very important and if the use of reminders can generate a significant proportion of extra data then it is a useful procedure. The reminder process is a viable approach not only for use with postal questionnaires, but also in computer based testing and integrated voice response methods. It should be noted that the best way to prevent the problems of missing data is to simply avoid it, by employing good data collection techniques and making an effort to chase up missing information. When the proportion of missing data becomes too large, no statistical technique will provide the solution.

## Conclusion

The first step in the analysis of incomplete data should involve quantifying the extent of missingness, identifying which individuals have missing data and at which assessments. In usual situations none of the missing QoL data are retrieved, and thus it is not possible to test formally a hypothesis that missingness is MAR as opposed to MNAR. Our study provided an example in which it was possible to carry out a formal test, confirming that data were MNAR and that simple imputation was unsatisfactory in this situation.

## Abbreviations

CIs: confidence intervals; DF: degrees of freedom; HSRU: Health Service Research Unit; LaN: last-and-next; LVCF: last value carried forwards; MAR: missing at random; MCAR: missing completely at random; MCS: mental component score; MNAR: missing not at random; NVCB: next value carried backwards; PCS: physical component score; PV: proportionate variance; QoL: quality of life; RCT: randomised controlled trial.

## Competing interests

The authors declare that they have no competing interests.

## Authors' contributions

SF analysed and interpreted the data, drafted the manuscript and gave final approval to the submitted manuscript. PMF conceived the idea, assisted in interpretation of the results, commented on drafts and gave final approval to the submitted manuscript. AM and GM were involved in the design and running of the RECORD trial including data collection, commented on drafts and gave final approval to the submitted manuscript. MKC was involved in the design and running of the RECORD trial, commented on drafts and gave final approval to the submitted manuscript.
